# HAT1 as a Lactyltransferase to Promote DNA Repair Through RPA1 Lactylation in Glioblastoma

**DOI:** 10.7150/ijbs.125306

**Published:** 2026-01-22

**Authors:** Yuzu Zhao, Jiang He, Zhiying Zhou, Tangmin Lai, Siwei Zeng, Nan Li, Yu He, Wei Zhou, Yongzhong Wu, Bo Xu

**Affiliations:** 1 Chongqing Key Laboratory of Intelligent Oncology for Breast Cancer, Intelligent Oncology Innovation Center Designated by the Ministry of Education, Chongqing University Cancer Hospital and Chongqing, University School of Medicine, Chongqing, China.; 2 Radiation Oncology Center, Chongqing University Cancer Hospital, Chongqing, China.; 3 Bioengineering College of Chongqing University, Chongqing, China.

**Keywords:** glioblastoma multiforme, Radioresistance, HAT1, Lactylation, RPA1

## Abstract

Glioblastoma multiforme (GBM) remains the most lethal primary brain tumor, owing to its aggressive nature and resistant to most of standard therapies, including radiation therapy. The glycolytic metabolic preference of cancer cells leads to the accumulation of lactate, a precursor for post-translational protein lactylation. While this modification is implicated in GBM, its mechanistic contribution to therapeutic resistance to radiation therapy is not well defined. Here, we identify histone acetyltransferase 1 (HAT1) as a candidate target for radiosensitization in GBM. We demonstrate that HAT1 acts as a lactyltransferase, directly catalyzing the lactylation of replication protein A1 (RPA1) at lysine 88 (K88). Furthermore, lactate drives the transcriptional upregulation of HAT1 via KAT2B-mediated lactylation of histone H4 at lysine 12 (H4K12la). Notably, lactate also induces auto-lactylation of HAT1 at lysine 15 (K15), which potentiates its enzymatic activity toward RPA1. Genetic ablation of HAT1 enhances radiotherapy efficacy *in vivo*. Correspondingly, targeted inhibition of RPA1 lactylation at K88, using a competitive peptide, reverses radioresistance in GBM cells. Collectively, these findings establish the lactate-driven HAT1-RPA1 lactylation axis as a critical regulator of radioresistance and nominate both HAT1 and lactylated RPA1 as novel therapeutic targets in GBM.

## Introduction

Glioblastoma (GBM) is a highly invasive and malignant primary brain tumor in adults^1^. According to the Central Brain Tumor Registry of the United States (CBTRUS), the five-year survival rate for GBM was 7.1% in the United States between 2017 and 2021^2^. Radiotherapy is a primary treatment for GBM^3^, ^4^, which eliminates tumor cells by inducing direct and indirect DNA damage^5^. However, comparing to sensitive tumors to radiotherapy, GBM is among the most radio-resistant tumors owing to its enhanced DNA repair activity^6^. As a result, patients derive limited benefit from postoperative radiotherapy due to this radioresistance. Simply escalating the radiation dose does not significantly improve survival and given the tumor's critical location, risks damaging adjacent normal brain tissues.^7^. Accumulating evidence indicates that inhibiting DNA damage repair (DDR) can overcome radioresistance in tumor cells^8^, ^9^. Therefore, targeting DNA repair pathways represents a promising therapeutic strategy for improving radiosensitivity in GBM.

Ionizing irradiation (IR) induces DNA double-strand breaks (DSBs), which are the most deleterious form of DNA damage^10^. These breaks are primarily repaired through two major pathways: non-homologous end joining (NHEJ) and homologous recombination (HR)^11^. NHEJ directly ligates broken DNA ends without a template, making it a rapid but error-prone mechanism. Repair initiates when the Ku heterodimer (Ku70/Ku80) recognizes and binds to the DSB ends. This recruits the DNA-dependent protein kinase catalytic subunit (DNA-PKcs), which phosphorylates downstream repair factors. The nuclease Artemis is then activated to process the damaged DNA ends. Finally, DNA ligase IV (LIG4) in complex with cross-complementing protein 4 (XRCC4) and XRCC4-like factor (XLF), catalyzes the ligation of the DNA termini^12^. In contrast, HR accurately repairs DSBs using a sister chromatid as a template. The MRN complex (MRE11-RAD50-NBS1), together with its co-factor CtIP binds to the DSB and initiates 5' end resection by nucleases such as EXO1 or DNA2^13^. The resulting 3' single-stranded DNA overhangs are rapidly coated by replication protein A (RPA). RPA is subsequently replaced by RAD51, which forms nucleoprotein filaments that mediate strand invasion into the homologous template, leading to displacement loop (D-loop) formation. The repair process is completed through the coordinated action of additional nucleases and ligases^14^.

Like many cancers, GBM cells prefer glycolysis to generate energy^15^. This metabolic shift leads to the excessive accumulation of lactate, an intermediate metabolite that promotes the malignant progression of GBM^16^. In recent years, lactylation induced by lactate is regarded as an emerging post-translational modification that is involved in numerous cancers. A growing body of evidence underscores its critical role in GBM pathogenesis and therapeutic response^17^-^19^. Notably, lactylation of key DDR regulators, including MRE11, NBS1, PARP1, and IFI16, has been implicated in driving therapeutic resistance^20^-^24^. Consequently, elucidating the mechanism by which protein lactylation governs radioresistance and identifying combination therapies to enhance radiosensitivity are anticipated to provide novel treatment avenues and improve survival for GBM patients.

Lactylation is a dynamic and reversible post-translational modification regulated by lactyltransferases ("writers"), delactylases ("erasers") and "readers"^25^, ^26^. Accumulating evidence indicates that several KAT family members including P300, CBP, KAT2A, KAT2B, KAT5, KAT7 and KAT8 have been identified as lactyltransferases^27^, ^28^. In this study, a systematic analysis of known "writers" and "erasers" across various tumors identified HAT1 as a promising biomarker in GBM. We demonstrate that HAT1 functions as a lactyltransferase, regulating proteome-wide protein lactylation, and confers radioresistance by promoting HR-mediated DDR. However, the substrates of lactylation, especially non-histone proteins remain largely unknown. Starting with the identification of HAT1 as a lactyltransferase, we focused on its role in regulating HR and discovered that HAT1 catalyzes the lactylation of RPA1. Moreover, this study further illustrated the underlying mechanism regulating HAT1 expression. We show that lactate promotes KAT2B-dependent lactylation of histone H4 at lysine 12 (H4K12) at the HAT1 promoter, thereby activating its transcription. Additionally, auto-lactylation of HAT1 at lysine 15 (K15) is indispensable for its lactyltransferase activity. In addition, peptides targeting RPA1 K88 lactylation were generated. This peptide showed significant inhibition of RPA1 lactylation and enhanced radiosensitivity. Taken together, our findings reveal that HAT1-driven RPA1 lactylation promotes HR-mediated radioresistance, indicating that targeting RPA1 lactylation might be a promising strategy to overcome radioresistance in GBM.

## Materials and Methods

### Reagents and antibodies

The Mycoplasma Detection Kit (#FM311-01) was obtained from TransGen Biotech (Beijing, China). For Immunohistochemistry (IHC), the rabbit reinforced polymer detection system (#PV-9001) and DAB substrate kit (#ZLI-9017) were supplied by ZSGB-BIO (Beijing, China). Beyotime Biotechnology (Shanghai, China) supplied Crystal violet staining solution (#C0121) for colony formation assay. The comet assay was conducted using Comet assay single cell gel electrophoresis kit (#4250-050-K) from Bio-Techne (Minneapolis, USA). NALA (#1614308) was obtained from Sigma-Aldrich. Lactyl-CoA (#HY-141540) was purchased from MedChemExpress (MCE, Shanghai, China). SimpleChIP Enzymatic Chromatin IP Kit (Magnetic Beads) (#9003) was obtained from Cell Signaling Technology (CST, Shanghai, China). Antibodies against HAT1 (#11432-1-AP), β-Tubulin (#10068-1-AP), GAPDH (#60004-1-Ig), β-actin (#66009-1-Ig), Flag (#66008-4-Ig) and HA (#51064-2-AP) were purchased from Proteintech (Wuhan, China). Pan-Kla (#PTM-1401RM), Acylation Antibody Sampler Kit Plus (#PTM-6680) including Pan-Kcr, Pan-Kbu, Pan-Ksu, Pan-Kglu, Pan-Kbz, Pan-Kpr, Pan-Kbhb, Pan-kma and Pan-Kac antibodies, L-Lactyl-Histone Antibody Sampler Kit ChIP grade (#PTM-7483) including H2A.ZK11la, H2BK15la, H2BK16la, H3K9la, H3K14la, H3K18la, H3K23la, H3K56la, H4K5la, H4K8la, H4K12la, H4K16la and H4 antibodies were purchased from PTM BIO (Hangzhou, China). γ-H2AX (#9718) and RPA1 (#2267) antibodies were purchased from Cell Signaling Technology (CST, Shanghai, China). For co-Immunoprecipitation, HA tag (C29F4) rabbit mAb (Magnetic bead conjugate) (#11846S) was purchased from CST (Shanghai, China). Anti-FLAG® M2 magnetic beads (#M8823) was obtained from Sigma Aldrich (Shanghai, China).

### Cell lines and cell culture

All cell lines used in this study were purchased from American Type Culture Collection (ATCC). The human GBM cell lines LN-229 and LN-18 along with human embryonic kidney cell line HEK-293T were cultured in DMEM medium supplemented with 10% fetal bovine serum (FBS) and 1% penicillin/streptomycin (P/S). All cell lines were cultured in the incubator with 5% CO_2_ and saturated humidity at 37 ℃. All cell lines used in this study were passaged fewer than 10 times. All cell lines were authenticated by short tandem repeat (STR). PCR was performed to detect mycoplasma contamination using the TransDetect PCR Mycoplasma Detection Kit.

### Analysis of patient data

Gene expression data of histone acetyltransferases (writers) and deacetylases (erasers) in patients with different types of tumors were obtained from GEPIA (http://gepia.cancer-pku.cn). The Log2(TPM + 1) values were automatically generated by the GEPIA website. LogFC(T/N) was calculated by subtracting the Log2(TPM + 1) values of normal tissues from the Log2(TPM + 1) values of tumor tissues in the corresponding cancer types. The expression of HAT1 in normal and tumor tissues of GBM and LGG was also generated from the GEPIA website, with a Log2FC Cutoff = 1 and a p-value Cutoff = 0.01. For the overall survival analysis of HAT1, the Kaplan-Meier Plotter was analyzed using the CGGA database (https://www.cgga.org.cn/), and the corresponding p-values were directly obtained from the database.

### IHC staining

This study was conducted in compliance with the principles of the Declaration of Helsinki. Informed consent was obtained from all the subjects. Ethics approval for human subjects was provided by the Ethics Committee of Chongqing University Cancer Hospital. IHC staining was performed according to a previous protocol^29^. Specifically, Paraffin-embedded samples were routinely sectioned at a thickness of 5 µm. The sections were deparaffinization in xylene and rehydration through a graded ethanol series. Following antigen retrieval, cell permeabilization and blockade of endogenous peroxidase, the sections were incubated with primary antibodies. Subsequently, the tissues were visualized using a rabbit reinforced polymer detection system and a DAB substrate kit, followed by nuclei staining. All images were obtained with microscopy.

### Plasmid construction

The sequence targeting HAT1 used for construction of lentiviral sgRNA was 5'-GCTACGCTCTTTGCGACCGT-3'. The sequences encoding Flag tagged HAT1, Flag tagged RPA1, HA tagged P300, HA tagged CBP, HA tagged HAT1, HA tagged KAT2A, HA tagged KAT2B, HA tagged KAT5, HA tagged KAT7, HA tagged KAT8 and His tagged RPA1 were amplified by PCR. PCR fragments were then cloned into pLV-Puro-CMV vector. RPA1 K88R mutant and HAT1 K15R mutant were constructed using the QuikChange Lightning Site-Directed Mutagenesis Kit.

### Western blot

Western blot was performed to detect the expression of proteins. In detail, cells were collected and lysed with RIPA lysis buffer. The proteins were separated by SDS-PAGE and transferred to PVDF membranes. The proteins were then probed with the indicated primary antibodies, followed by incubation with corresponding secondary antibodies. The immunoblots were visualized using a ChemiDoc XRS+ system.

### Immunofluorescence (IF)

IF staining was performed to investigate the foci of γ-H2AX or RPA1 in GBM cells. In detail, cells were counted and seeded onto coverslips in 12-well plates. After IR, cells were collected at different time points (0 h, 1 h and 24 h). The cells were then fixed, permeabilized, and blocked to prevent nonspecific binding. Subsequently, the cells were incubated with γ-H2AX or RPA1 antibodies, followed by the corresponding fluorescent secondary antibodies. The coverslips were sealed with an anti-fade regent containing DAPI. γ-H2AX-positive or RPA1-positive cells in random fields were imaged using a Leica Stellaris 5 confocal fluorescence microscope.

### Comet assays

Comet assays were conducted to detect the DNA damage. Specifically, cells were seeded in 60 mm culture dishes and subjected to IR. cells were collected at the indicated time points (0 h, 1 h and 24 h), diluted to a density of 1 × 10^6^ cells /ml, and mixed with molten LMAgarose at a ratio of 1:10 (V/V). The mixture was immediately added onto comet slides. After gelling, the slides were subjected to lysis and electrophoresis, the cells were stained with goldview and visualized by fluorescence microscopy.

### Colony formation assays

Colony formation assays were conducted to detect the cellular radiosensitivity. Cells in the logarithmic phase were counted and seeded in 6-well plates. After IR at specified doses (0 Gy, 2 Gy, 4 Gy, 6 Gy and 8 Gy). Irradiated cells were cultured under standard condition for about 2 weeks. Once obvious colonies had formed, colonies were fixed, stained and imaged.

### HR and NHEJ reporter assays

The efficiencies of homologous recombination (HR) and non-homologous end joining (NHEJ) were assessed using the DR-GFP and EJ5-GFP reporter systems, respectively. Briefly, the indicated cells expressed DR-GFP or EJ5-GFP were transfected with I-SceI plasmids. After 48 h, cells were collected and analyzed by flow cytometry, The HR or NHEJ efficiency was quantified based on the percentage of GFP-positive cells.

### 4D label-free lactylation quantitative proteomics and analysis

4D label-free lactylation proteomics in LN-229 cells was performed by Shanghai Bioprofile Technology.

### *In vitro* lactylation assays

HA-tagged HAT1 and His-tagged RPA1 proteins were co-incubated in reaction buffer (50 mM HEPES, pH7.8, 30 mM KCl, 0.25 mM EDTA, 5.0 mM MgCl2, 5.0 mM sodium butyrate, 2.5 mM DTT) at 30 °C for 30 min with 20 mM lactyl-CoA. The reaction was terminated by adding 5× SDS sample buffer and heating at 95 °C  for 5 min. The expression of Pan-Kla, Pan-ac, HAT1 and RPA1 was detected by Western blot.

### DNA-protein binding assays

For DNA-protein binding assays, biotin-labeled ssDNA was synthesized. HEK-293T cells were transfected with plasmids encoding Flag-tagged RPA1 or RPA1 K88R mutant. The cells were collected and lysed in binding buffer (10 mM Tris-HCl, PH 7.5, 100 mM NaCl, 10 g/ml BSA 10% glycerol and 0.5% NP-40). Whole cell lysates were then incubated with biotin-conjugated ssDNA at room temperature for 30 min. Streptavidin beads (S beads) were added to the ssDNA-lysates mixture at room temperature for 1 h to pull down the bound proteins. The ssDNA-binding proteins were analyzed by Western blot.

### Chromatin immunoprecipitation (ChIP)-qPCR

ChIP was performed using a SimpleChIP Enzymatic Chromatin IP Kit according to the manufacturer's instructions. The immunoprecipitated DNA was purified and evaluated using qPCR. The primers used to amplify various regions of the HAT1 promoter were listed in Supplementary Table S1.

### Orthotopic models

All animal studies were conducted in accordance with the relevant guidelines of the Ethics Committee of Chongqing University Cancer Hospital, following the National Institutes of Health Guidelines for animal welfare. 4-week-old BALB/c nude mice were housed in ICV system for about 1-2 weeks. Under tribromoethanol anesthesia (0.2 mL/10 g body weight), mice were intracranially injected with 1 × 10⁵ LN-229 cells (in 10 μL PBS) at coordinates 2 mm lateral and 1 mm anterior to the bregma, at a depth of 3.5 mm below the dura. About 2 weeks after the injection, when Luciferase labeled tumor appeared. Each group of mice was divided into two groups randomly, one received localized radiotherapy (5 Gy IR, 2 fractions with non-tumor areas shielded), while the other served as the non-irradiated control. Tumor growth was monitored with the Lumina Series. At the end of the experiment, all mice were humanely euthanized using carbon dioxide asphyxiation.

### Statistical analysis

GraphPad Prism software (version 9.0) was used for statistical analyses. Quantitative data are presented as the mean ± SD. Two-way analysis of variance (ANOVA) or Student's t test were used to assess significant differences. P-values of < 0.05 (*), P-values of < 0.01 (**) and P-values of < 0.001 (***) were considered statistically significant.

## Results

### HAT1 expression correlates with glioma grade and poor prognosis

**Figure 1:**
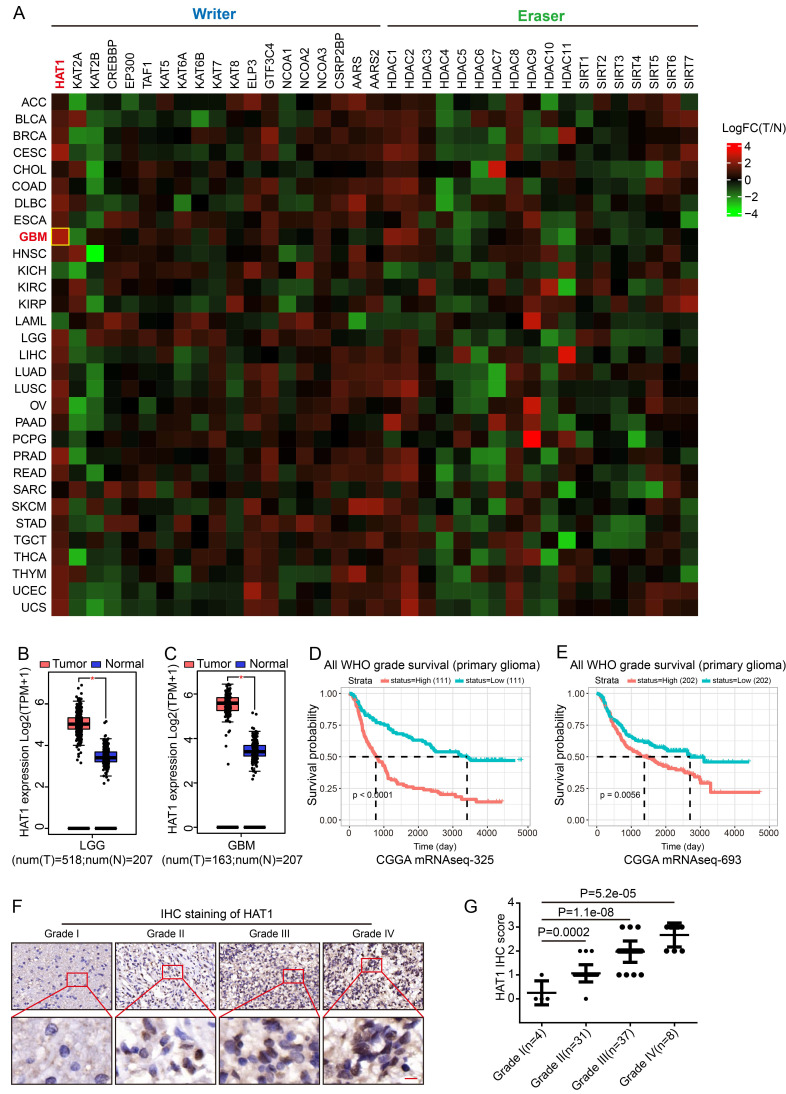
** HAT1 expression correlates with glioma grade and poor prognosis. (A).** Analysis of known lactylation “writers” and “erasers” expression across various tumors. **(B).** Expression levels of HAT1 in normal tissues compared with low-grade glioma (LGG) samples. **(C).** Expression levels of HAT1 in normal tissues compared with GBM samples. **(D).** Correlation between HAT1 expression and patient prognosis in the CGGA mRNAseq-325 dataset. **(E).** Correlation between HAT1 expression and patient prognosis in the CGGA mRNAseq-693 dataset. **(F).** Representative IHC images of HAT1 staining in human glioma specimens (Grade I-IV). **(G).** Statistical analyses of HAT1 staining intensity from panel F. All data were analyzed using two-way analysis of variance (ANOVA).

The glycolytic metabolism of cancer cells results in the accumulation of lactate, which drives enhanced protein lactylation. To identify key regulators of lactylation, we analyzed the expression of known lactylation "writers" and "erasers" across tumor types. HAT1 showed the highest expression level in GBM samples (Fig. 1A and Supplementary Table S2) and was broadly elevated in several cancers compared to normal tissues (Supplementary Fig. S1A). Specifically, HAT1 levels were higher in low-grade glioma (LGG) (Fig. 1B) and GBM (Fig. 1C and Supplementary Fig. S1B) relative to normal brain. Elevated HAT1 expression correlated significantly with poor patient prognosis (Fig. 1D, 1E and Supplementary Fig. S1C). IHC further revealed that HAT1 expression increased with glioma grade (Fig. 1F and 1G). These findings establish HAT1 as a potential biomarker for glioma grading and prognosis.

### HAT1 is required for the global lactylation in GBM cells

**Figure 2:**
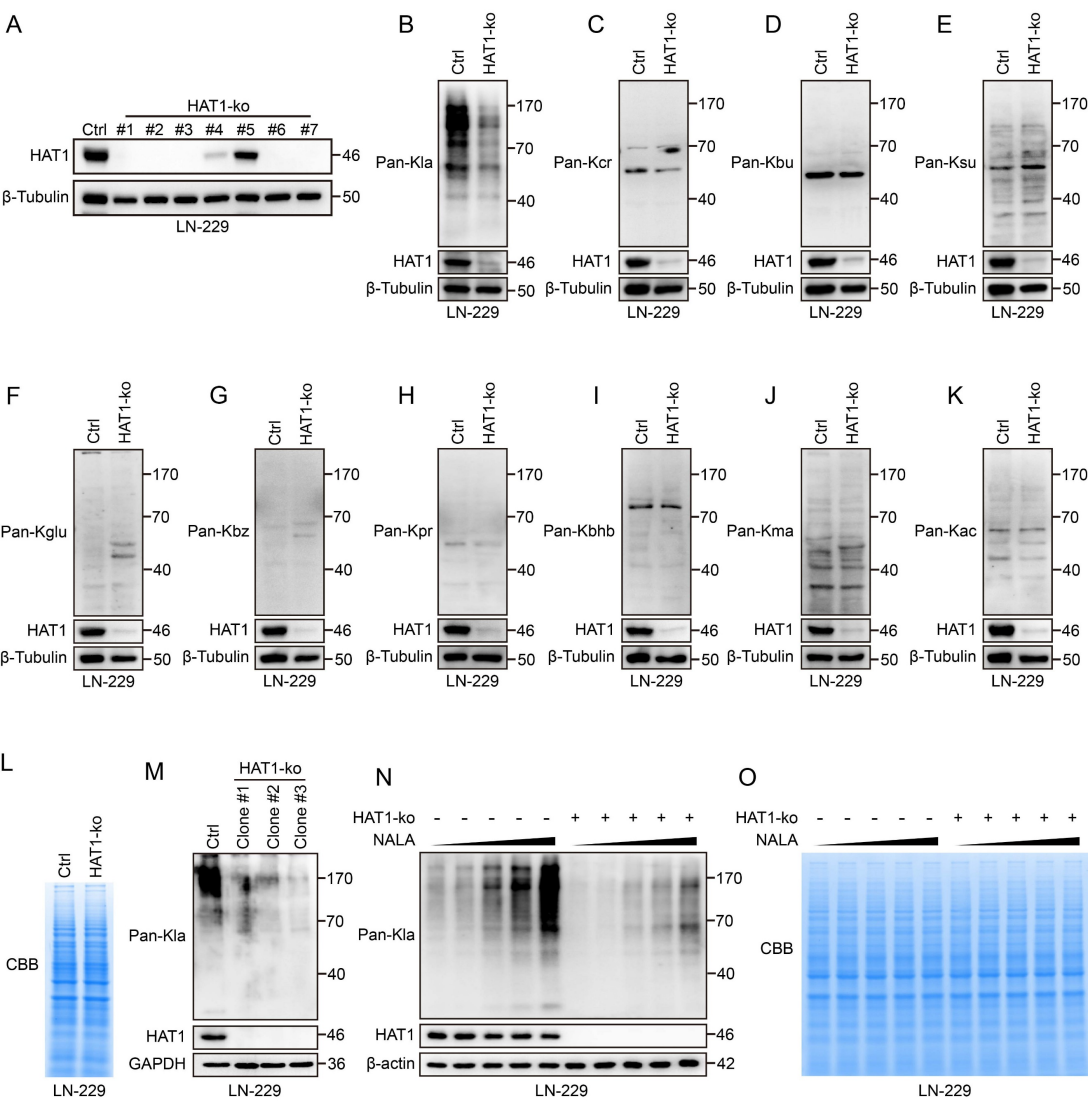
** HAT1 is required for global lactylation in GBM cells. (A).** Immunoblot analysis of HAT1 in HAT1-ko LN-229 monoclonal cells. **(B).** The level of pan L-Lactyl Lysine (Pan-Kla) in HAT1-ko cells. **(C).** The expression of pan Crotonyllysine (Pan-Kcr) in HAT1-ko cells. **(D).** The level of pan Butyryllysine (Pan-Kbu) in HAT1-ko cells. **(E).** The expression of global Succinyllysine (Pan-Ksu) in HAT1-ko cells. **(F).** The level of global Glutaryllysine (Pan-Kglu) in HAT1-ko cells. **(G).** The expression of global Benzoyllysine (Pan-Kbz) in HAT1-ko cells. **(H).** The level of pan Propionyllysine (Pan-Kpr) in HAT1-ko cells. **(I).** The expression of global β-Hydroxybutyryllysine (Pan-Kbhb) in HAT1-ko cells. **(J).** The level of global Malonyllysine (Pan-Kma) in HAT1-ko cells. **(K).** The expression of global Acetyllysine (Pan-Kac) in HAT1-ko cells. **(L).** Coomassie brilliant blue staining of total proteins from control and in HAT1-ko cells. **(M).** The expression of pan-Kla and HAT1 in multiple HAT1-ko monoclonal cells. **(N).** NALA induced pan-Kla in HAT1-ko cells. **(O).** Coomassie brilliant blue staining of total proteins from NALA-treated control and HAT1-ko cells.

To further explore the functional role of HAT1 in GBM, HAT1 was knocked out in GBM cell lines LN-229 and LN-18 (Fig. 2A and Supplementary Fig. S2A). Since HAT1 is known as an acyltransferase, we profiled 10 different acylation modifications in HAT1-knockout GBM cells. The results showed that the L-lactylation of global proteins decreased in both HAT1-ko LN-229 and LN-18 cells (Fig. 2B and Supplementary Fig. S2B). Whereas, another 9 types of acylation modifications including Crotonyllysine (Fig. 2C and Supplementary Fig. S2C), Butyryllysine (Fig. 2D and Supplementary Fig. S2D), Succinyllysine (Fig. 2E and Supplementary Fig. S2E), Glutaryllysine (Fig. 2F and Supplementary Fig. S2F), Benzoyllysine (Fig. 2G and Supplementary Fig. S2G), Propionyllysine (Fig. 2H and Supplementary Fig. S2H), β-Hydroxybutyryllysine (Fig. 2I and Supplementary Fig. S2I), Malonyllysine (Fig. 2J and Supplementary Fig. S2J) and Acetyllysine (Fig. 2K and Supplementary Fig. S2K) were not regulated by HAT1. Coomassie brilliant blue (CBB) staining further revealed equivalent protein loading between control and HAT1-ko cell lysates (Fig. 2L). Consistent with these results, the lactylation of global proteins decreased significantly in monoclonal LN-229 cells with HAT1 knockout (Fig. 2M). Numerous studies have proved that sodium lactate (NALA) induces the lactylation of proteins^20^, ^25^. We therefore treated LN-229 cells with gradient concentrations of NALA (0 mM, 1 mM, 5 mM, 10 mM and 50 mM) to induce protein lactylation. The results showed that the lactylation was elevated by NALA in a gradient-dependent manner. Besides, knocking out of HAT1 decreased the lactylation of global proteins (Fig. 2N). CBB staining showed equivalent protein loading among all cells. (Fig. 2O). These results indicate that HAT1 is required for protein lactylation in GBM cells.

### HAT1 promotes DDR and confers radioresistance in GBM cells by HR pathway

**Figure 3:**
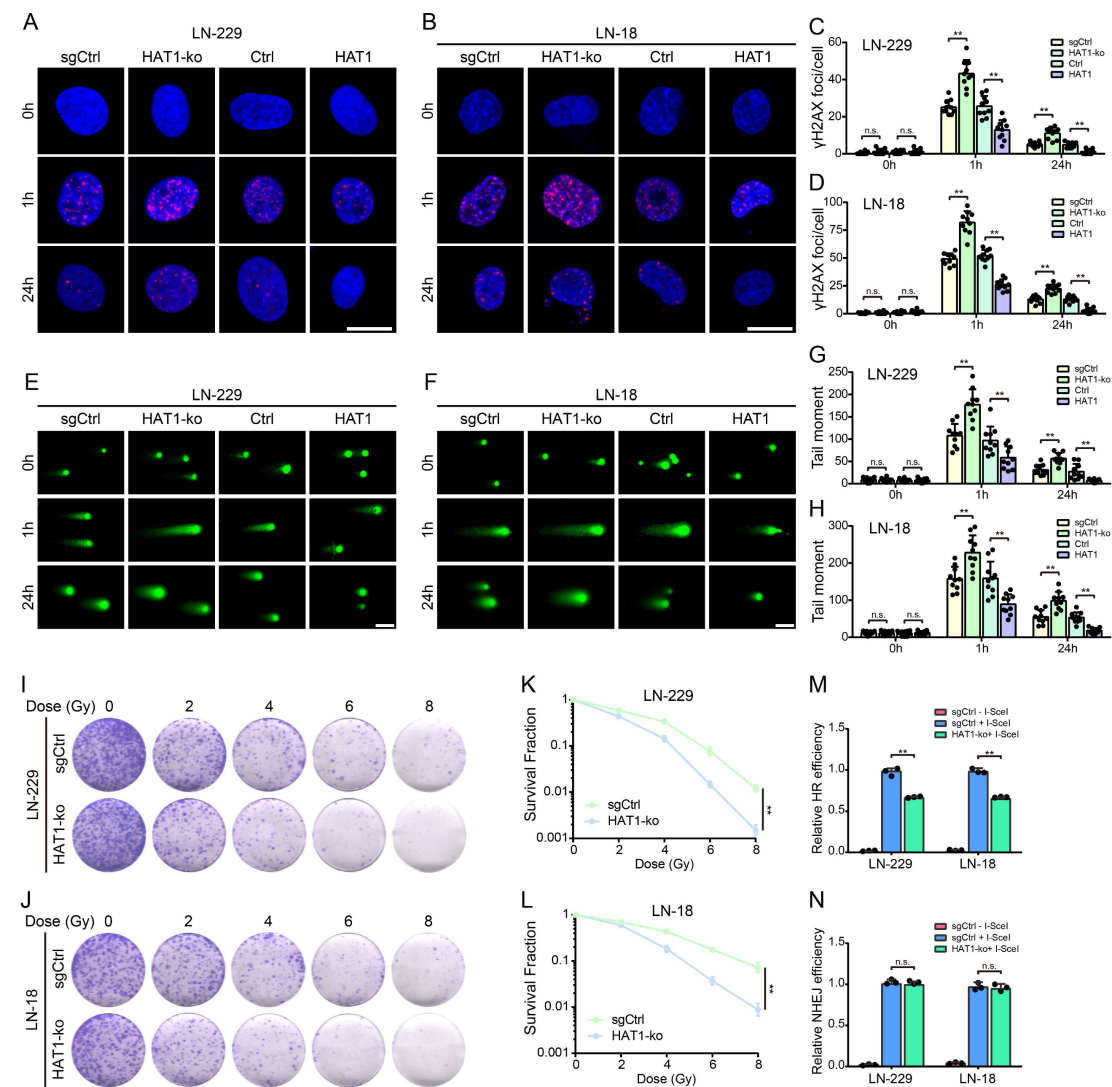
** HAT1 promotes DDR and confers radioresistance in GBM cells by HR pathway. (A).** The foci of γ-H2AX in HAT1-ko and HAT1 overexpressing LN-229 cells, scale bar, 10μm.** (B).** The foci of γ-H2AX in HAT1-ko and HAT1 overexpressing LN-18 cells, scale bar, 10μm. **(C).** Quantification of γ-H2AX foci in panel A. **(D).** Quantification of γ-H2AX foci in panel B. **(E).** Tailed DNA in HAT1-ko and HAT1 overexpressing LN-229 cells, scale bar, 100μm. **(F).** Tailed DNA in HAT1-ko and HAT1 overexpressing LN-18 cells, scale bar, 100μm. **(G).** Quantification of tailed DNA in panel E. **(H).** Quantification of tailed DNA in panel F.** (I).** The clonogenic ability of HAT1-ko LN-229 cells treated with the indicated doses of IR.** (J).** The clonogenic ability of HAT1-ko LN-18 cells treated with the indicated doses of IR. **(K).** Quantification of colony formation in panel I. **(L).** Quantification of colony formation in panel J. **(M).** HR efficiency in HAT1-ko GBM cells. **(N).** NHEJ efficiency in HAT1-ko GBM cells. All data were analyzed using two-way analysis of variance (ANOVA), **P < 0.01.

To further investigate the role of HAT1 in DDR, IF staining of γ-H2AX, comet assays and colony formation assays were conducted in HAT1-ko and HAT1-overexpressing GBM cells, respectively (Supplementary Fig. S3A and S3B). As a universal marker of DNA damage, γ-H2AX foci persisted longer in HAT1-ko cells (LN-229 and LN-18) compared to controls, while HAT1 overexpression shortened their persistence (Fig. 3A-3D). In addition, Western blot analysis of γ-H2AX levels further verified that HAT1 depletion prolonged DNA damage signaling (Supplementary Fig. S3C and S3D). Comet assays revealed increased DNA damage (longer comet tails) in HAT1-ko cells and reduced damage in HAT1-overexpressing cells (Fig. 3E-3H). Colony formation assays demonstrated that HAT1 depletion significantly enhanced cellular radioresensitivity in GBM cells (Fig. 3I-3L). Based on DR-SceGFP and EJ5-SceGFP reporter systems, we found that HR efficiency decreased significantly in HAT1-ko cells (Fig. 3M), whereas NHEJ efficiency remained unaffected (Fig. 3N). Consistent with HR impairment, RPA1 foci, a key HR mediator, persisted longer in HAT1-ko cells and resolved faster upon HAT1 overexpression (Supplementary Fig. S3E-3H). Collectively, these findings demonstrate that HAT1 facilitates DDR, particularly HR, in GBM cells and identify HAT1 as a potential therapeutic target to increase radiosensitivity.

### HAT1 mediated RPA1 lactylation promotes HR and radioresistance in GBM cells

**Figure 4:**
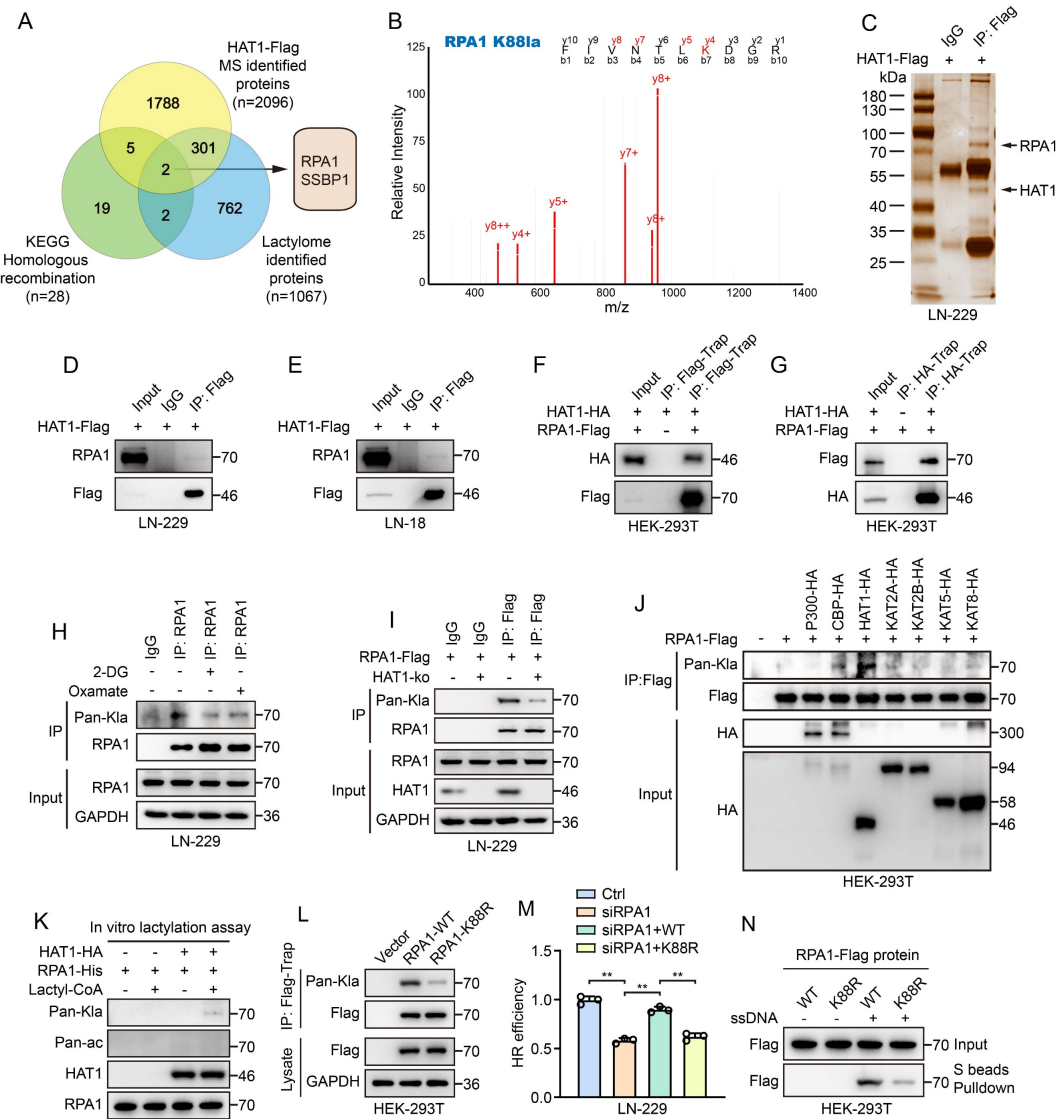
** HAT1 mediated RPA1 lactylation promotes HR and radioresistance in GBM cells. (A).** Venn diagram showing the intersection among lactylated proteins, HAT1 interacting proteins, and 28 KEGG enriched HR genes.** (B).** Secondary mass spectrometry spectrum identifying lactylation at the K88 site of RPA1. **(C).** Silver staining of HAT1 interacting proteins. **(D).** Interaction between HAT1 and RPA1 in LN-229 cells. **(E).** Interaction between HAT1 and RPA1 in LN-18 cells. **(F, G).** Interaction between exogenously expressed HAT1 and RPA1 in HEK-293 cells. **(H).** RPA1 lactylation in cells treated with glucose analog 2-DG and LDH-A inhibitor oxamate. **(I).** RPA1 lactylation in HAT1-ko cells. **(J).** RPA1 lactylation in cells overexpressing with 7 acyltransferases including P300, CBP, HAT1, KAT2A, KAT2B, KAT5 and KAT8. **(K).** *In vitro* lactylation assays showing lysine lactyltransferase activity of HAT1 toward RPA1. **(L).** The levels of lactylated RPA1 in RPA1 K88R mutant cells. **(M).** HR efficiency in RPA1 K88R mutant cells. **(N).** The level of RPA1 interacted ssDNA in RPA1 K88R mutant cells.

Previous findings demonstrated that HAT1 regulates global protein lactylation in GBM cells. To further elucidate the role of HAT1-driven lactylation in DDR, we performed 4D label-free lactylation proteomics and identified 1,067 lactylated proteins in GBM cells. In parallel, we performed high-resolution liquid chromatography-tandem MS (LC-MS/MS) analysis of Flag-tagged HAT1 and detected 2,096 HAT1-interacting proteins (Supplementary Table S3). Since HAT1 regulates HR efficiency in GBM cells, we intersected these datasets with a KEGG-enriched HR gene set (28 genes; Supplementary Table S4). The analysis indicated that RPA1 and SSBP1 as potential lactylated substrates of HAT1 that promote HR-mediated DDR in GBM cells (Fig. 4A). RPA1 is a core component of the HR machinery, yet the role of its lactylation in regulating HR remains unclear. Furthermore, lactylation proteomics revealed K88 as a lactylated site on RPA1 in LN-229 cells (Fig. 4B). Silver staining further indicated the interaction between HAT1 and RPA1 (Fig. 4C), which was validated by co-immunoprecipitation in both LN-229 (Fig. 4D) and LN-18 cells (Fig. 4E). Consistent with this, exogenous HAT1 and RPA1 also interacted when co-expressed in HEK-293T cells (Fig. 4F and 4G). Treatment of LN-229 cells with both the glucose analog 2-deoxy-d-glucose (2-DG) and lactate dehydrogenase-A (LDH-A) inhibitor oxamate inhibited RPA1 lactylation (Fig. 4H), as did depletion of HAT1 (Fig. 4I). Among 7 acyltransferases (p300, CBP, HAT1, KAT2A, KAT2B, KAT5, and KAT8), only HAT1 increased RPA1 lactylation (Fig. 4J). An *in vitro* lactylation assay further confirmed that HAT1 directly lactylates RPA1 (Fig. 4K). To determine the functional lactylation site, we generated an RPA1 K88R mutant. Lactylation was markedly reduced in cells expressing RPA1 K88R (Fig. 4L), and HR efficiency was impaired compared to wild-type controls (Fig. 4M). Using biotin-labeled ssDNA in DNA-protein binding assay, we observed attenuated ssDNA-binding affinity of the RPA1 K88R mutant (Fig. 4N). Colony formation assays further confirmed that RPA1 K88 lactylation promotes radioresistance in GBM cells (Supplementary Fig. S4). Collectively, these findings demonstrate that HAT1 promotes HR-mediated radioresistance by lactylating RPA1 at K88.

### Lactate promotes the expression of HAT1 and its auto-Lactylation to promote HR

**Figure 5:**
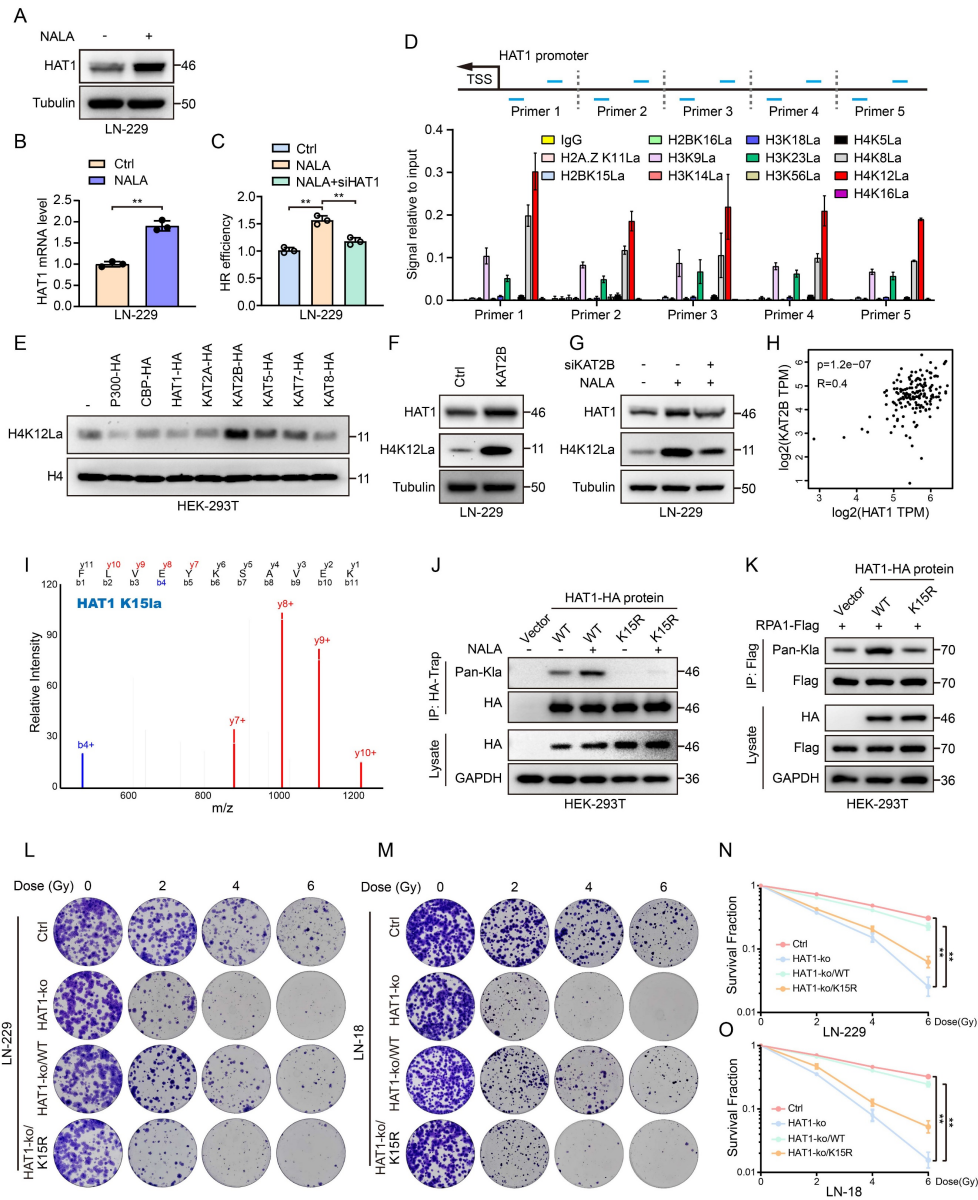
** Lactate promotes the expression of HAT1 and its auto-Lactylation to promote HR. (A).** The expression of HAT1 in LN-229 cells with NALA treatment.** (B).** The mRNA level of HAT1 in LN-229 cells with NALA treatment. **(C).** HR efficiency in LN-229 cells treated with NALA or knocking down of HAT1. **(D).** enrichment of histone lactylation at different sites including H2AZ. K11, H2BK15, H2BK16, H3K9, H3K14, H3K18, H3K23, H3K56, H4K5, H4K8, H4K12 and H4K16 at the HAT1 promoter. **(E).** The level of lactylated H4K12 in HEK-293T cells overexpressing the indicated acyltransferases (P300, CBP, HAT1, KAT2A, KAT2B, KAT5, KAT7 and KAT8). **(F).** The expression of HAT1 and lactylated H4K12 in LN-229 cells overexpressing KAT2B. **(G).** HAT1 expression and lactylated H4K12 in LN-229 cells treated with NALA or knocking down of KAT2B. **(H).** Correlation analysis between HAT1 and KAT2B. **(I).** Secondary mass spectrometry spectrum identifying lactylation at the K15 site of HAT1. **(J).** HAT1 auto-lactylation in HAT1 K15R mutant cells. **(K).** RPA1 lactylation in HAT1 K15R mutant cells. **(L).** The clonogenic ability of HAT1 K15R mutant LN-229 cells treated with IR. **(M).** The clonogenic ability of HAT1 K15R mutant LN-18 cells treated with IR. **(N).** Quantification of colony formation in panel L. **(O).** Quantification of colony formation in panel M. All data were analyzed using two-way analysis of variance (ANOVA), **P < 0.01.

Lactylation proteomics identified lactylation at the 15_th_ lysine (K15) of HAT1 in GBM cells, indicating that HAT1 itself undergoes lactylation. Treatment of GBM cells with 10 mM NALA increased both HAT1 protein (Fig. 5A) and mRNA levels (Fig. 5B). HR efficiency analysis further indicated that NALA promotes HR in a HAT1-dependent manner (Fig. 5C). Histone lactylation links lactate accumulation to functional alterations in tumors. To explore how HAT1 expression is regulated, we performed ChIP-qPCR to assess enrichment of various lactylated histone marks at the HAT1 promoter, including H2A.ZK11, H2BK15, H2BK16, H3K9, H3K14, H3K18, H3K23, H3K56, H4K5, H4K8, H4K12 and H4K16. All tested sites showed enrichment, with H4K12 lactylation (H4K12la) exhibiting the strongest interaction (Fig. 5D). Among eight overexpressed acyltransferases (p300, CBP, HAT1, KAT2A, KAT2B, KAT5, KAT7, and KAT8) in HEK-293T cells, KAT2B significantly enhanced H4K12la (Fig. 5E). Overexpression of KAT2B in GBM cells increased both HAT1 expression and H4K12la levels (Fig. 5F). while KAT2B knockdown reversed the NALA-induced upregulation of HAT1 and H4K12la (Fig. 5G). Correlation analysis further supported a positive relationship between HAT1 and KAT2B (Fig. 5H). These findings indicate that lactate elevates HAT1 expression and H4K12la via KAT2B, which promotes the accumulation of H4K12la at the HAT1 promoter to drive transcription. According to the results of lactylation proteomics, K15 site of HAT1 was lactylated in GBM cells (Fig. 5I). Mutation of K15 to arginine (K15R) abolished HAT1 auto-lactylation (Fig. 5J) and reduced RPA1 lactylation (Fig. 5K), demonstrating that the lactyltransferase activity of HAT1 critically depends on its auto-lactylation at K15. Colony formation assays further confirmed that HAT1 K15 lactylation promotes radioresistance in GBM cells (Fig. 5L-5O). Together, these results establish that lactate-driven, KAT2B-mediated H4K12la upregulates HAT1 expression, and that subsequent auto-lactylation of HAT1 at K15 is essential for its activity in promoting HR.

### Targeting HAT1 and RPA1 K88 lactylation enhance radiosensitivity in GBM cells

**Figure 6:**
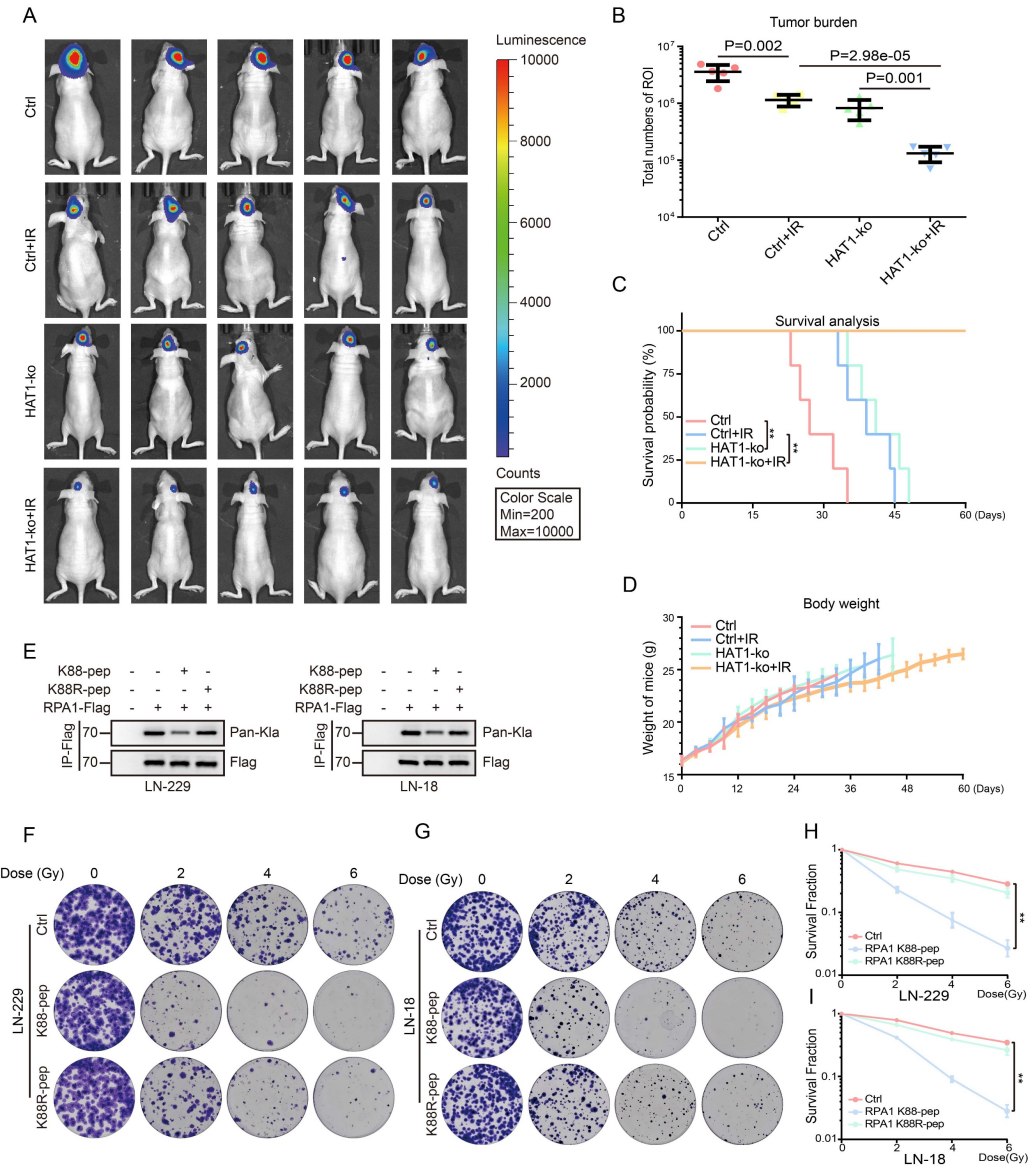
** Targeting HAT1 and RPA1 K88 lactylation regulate radiosensitivity in GBM cells. (A).** *In vivo* imaging of luciferase-labeled intracranial GBM xenografts in the indicated groups.** (B).** Quantification of bioluminescent signals from panel A.** (C).** Overall survival of mice bearing intracranial xenografts across the indicated groups.** (D).** Body weight of the indicated groups of mice.** (E).** RPA1 lactylation in GBM cells treated with RPA1 K88 specific peptides. **(F).** The clonogenic ability of LN-229 cells treated with RPA1 K88 specific peptides and IR. **(G).** The clonogenic ability of LN-18 cells treated with RPA1 K88 specific peptides and IR. **(H).** Quantification of colony formation in panel F. **(I).** Quantification of colony formation in panel G. All data were analyzed using two-way analysis of variance (ANOVA), **P < 0.01.

Given that HAT1 promotes RPA1 lactylation to drive HR-mediated radioresistance, we employed luciferase-labeled intracranial xenograft models to evaluate the therapeutic potential of inhibiting this axis. HAT1 knockout alone inhibited tumorigenesis *in vivo*, and its combination with IR significantly suppressed tumor growth more effectively than either treatment alone (Fig. 6A and 6B). Mice receiving combined HAT1 depletion and IR also exhibited prolonged overall survival (Fig. 6C), with no significant differences in body weight observed across groups (Fig. 6D). To specifically disrupt RPA1 K88 lactylation, peptides that specific target K88 site of RPA1 (RPA1 K88-pep: RFIVNTLKDGRRVVI) and RPA1 K88R mutant peptide (RPA1 K88R-pep: RFIVNTLRDGRRVVI) were generated (Fig. 6E). As expected, treatment with RPA1 K88-pep, but not the mutant peptide, significantly reduced colony formation in LN-229 cells (Fig. 6F-6I). These results demonstrate that targeting either HAT1 or RPA1 K88 lactylation effectively sensitizes GBM to radiotherapy.

## Discussion

GBM is the most common and aggressive primary brain tumor in adults. Mutations of isocitrate dehydrogenase (IDH) enzymes drive tumorigenesis, suggesting the central role of metabolic dysregulation in GBM pathogenesis^30^. The Warburg effect describes the glycolytic preference of cancer cells, which leads to elevated glucose uptake and lactate accumulation regardless of oxygen availability^31^, ^32^. Beyond serving as an energy source, lactate has recently been recognized as a substrate for protein lactylation, a dynamic post-translational modification^25^, ^27^. Emerging evidence highlight lactylation as a promising therapeutic target in GBM^17^, ^19^. Here, we identified HAT1 as an essential acyltransferase that mediates protein lactylation in GBM. We demonstrate that HAT1 served as a lactyltransferase to promote the lactylation of RPA1. This study further demonstrated a lactate-driven signaling axis: KAT2B-H4K12la-HAT1-RPA1la that promotes HR-mediated DDR and radioresistance in GBM. Furthermore, a peptide that specifically targets RPA1 K88 lactylation significantly enhanced radioresensitivity in GBM cells when combined with IR. Collectively, our study illustrates that the fresh role of HAT1 as a lactyltransferase regulating protein lactylation to promote DDR and radioresistance. Peptide that specifically targets RPA1 K88 lactylation as a radiosensitizing strategy for GBM.

Lactylation was initially characterized on histones, with identified sites including H3K4, H3K9, H3K14, H3K18, H4K5 and H4K12^33^. Among these, H4K12la, induced by lactate, activates RAD23A expression to enhance DDR and confer niraparib resistance in ovarian cancer^34^. Recent studies have expanded this landscape, revealing lactylation on diverse non-histone proteins such as YY1^35^, HMGB1^36^, Snail1^37^, fatty acid synthase^38^, ulk1^39^, METTL16^40^, Mecp2^41^, Nucleolin^42^ and p53^43^. Lactylation of these non-histone proteins regulates diverse biological processes, including the DDR^44^, ^45^. Notably, lactylation of the key DDR components contributes to therapeutic resistance across multiple cancers. In detail, MRE11 lactylation promotes HR and confers chemoresistance in cancer cells^20^. NBS1, another component of the MRN complex, is also lactylated driven by lactate, which promotes HR-mediated DNA repair and chemotherapy resistance^21^. Besides, lactylation of XRCC1 confers therapeutic resistance in ALDH1A3-overexpressing GBM^17^. Formaldehyde induced PARP1 lactylation promotes DNA repair and tumor cell growth^22^. PARP1 lactylation regulates its ADP-ribosylation activity to facilitate DNA repair^23^. Other proteins, including METTL3 and IFI16, have also been linked to the DDR through lactylation^24^, ^46^. In this study, our findings revealed that HAT1 promoted radioresistance through HR pathway but not NHEJ pathway. As a core HR component, RPA1 is essential for this process, yet the role of RPA1 lactylation remains unexplored. Elucidating RPA1 lactylation and its underlying mechanism is therefore critical for understanding the broader impact of lactylation in DDR.

Interestingly, HAT1 expression was elevated following NALA treatment, suggesting that lactylation regulates HAT1 expression. We further found that the lactylation at 12 histone sites, including H2A.Z K11, H2K15, H2K16, H3K9, H3K14, H3K18, H3K23, H3K56, H4K5, H4K8, H4K12 and H4K16 was enriched at HAT1 promoter, with H4K12la showing the strongest accumulation. Among several KAT family members tested, KAT2B, KAT5 and KAT7 enhanced H4K12la, with KAT2B having the most pronounced effect. This study further demonstrated that lactate increases H4K12 lactylation via KAT2B. However, whether other histone lactylation sites, H2A.Z K11la, H2K15la, H2K16la, H3K9la, H3K14la, H3K18la, H3K23la, H3K56la, H4K5la, H4K8la and H4K16la regulate HAT1 expression and whether other KAT family members, KAT5 and KAT7 regulate H4K12la or histone lactylation at other histone sites still need further investigation.

It is noteworthy that auto-lactylation of HAT1 at K15 is also found in lactylation proteomics in GBM cells. According to previous studies, auto-acetylation is required for the acetyltransferase activity of several KATs, such as P300, KAT5 (Tip60), KAT7 (MYST) and KAT8 (hMOF)^47^-^50^. However, the requirement of auto-lactylation for the lactyltransferase activity was unknown. This study illustrated that HAT1 K15 was required for its auto-lactylation and for RPA1 lactylation. Mutation of HAT1 K15 inhibited RPA1 lactylation, and reduced clonogenic survival, establishing that auto-lactylation is critical for HAT1's lactyltransferase function.

In summary, our data demonstrates that HAT1 functions as an emerging lactyltransferase to promote HR-mediated radioresistance in GBM. In addition, this study illustrates the lactate induced KAT2B-H4K12la-HAT1-RPA1la signaling axis. Furthermore, both HAT1 knockout and a peptide specifically targeting RPA1 K88 provide new avenues for combination therapy in GBM.

## Supplementary Material

Supplementary figures.

Supplementary table 1.

Supplementary table 2.

Supplementary table 3.

Supplementary table 4.

## Abbreviations

GBM: Glioblastoma
HR: homologous recombination
NHEJ: non-homologous end joining
CBTRUS: The Central Brain Tumor Registry of the United States
DDR: DNA damage repair
IHC: Immunohistochemistry
IF: Immunofluorescence
Chromatin immunoprecipitation: ChIP
Kcr: Crotonyllysine
Kbu: Butyryllysine
Ksu: Succinyllysine
Kglu: Glutaryllysine
Kbz: Benzoyllysine
Kpr: Propionyllysine
Kbhb: β-Hydroxybutyryllysine
Kma: Malonyllysine
Kac: Acetyllysine
Kla: L-lactylation
CBB: Coomassie brilliant blue
NALA: Sodium lactate
DSBs: DNA double-strand breaks
LC-MS/MS: High-resolution liquid chromatography-tandem
2-DG: 2-deoxy-d-glucose
LDH-A: lactate dehydrogenase-A
IDH: Isocitrate dehydrogenase
DNA-PKcs: DNA-dependent protein kinase catalytic subunit
LIG4: DNA ligase IV
XRCC4: Cross-complementing protein 4
XLF: XRCC4-like factor
MRN: MRE11-RAD50-NBS1
RPA: Replication protein A
D-loop: Displacement loop
