# Supplementary material for: HAT1 as a Lactyltransferase to Promote DNA Repair Through RPA1 Lactylation in Glioblastoma
Source: Int J Biol Sci. 2026 Jan 22;22(4):1906–19. doi: 10.7150/ijbs.125306 (PMC12905578; doi:10.7150/ijbs.125306)
Supplement: Supplementary file 1 — Supplementary figures. [file ijbsv22p1906s1.pdf]

Fig.S1

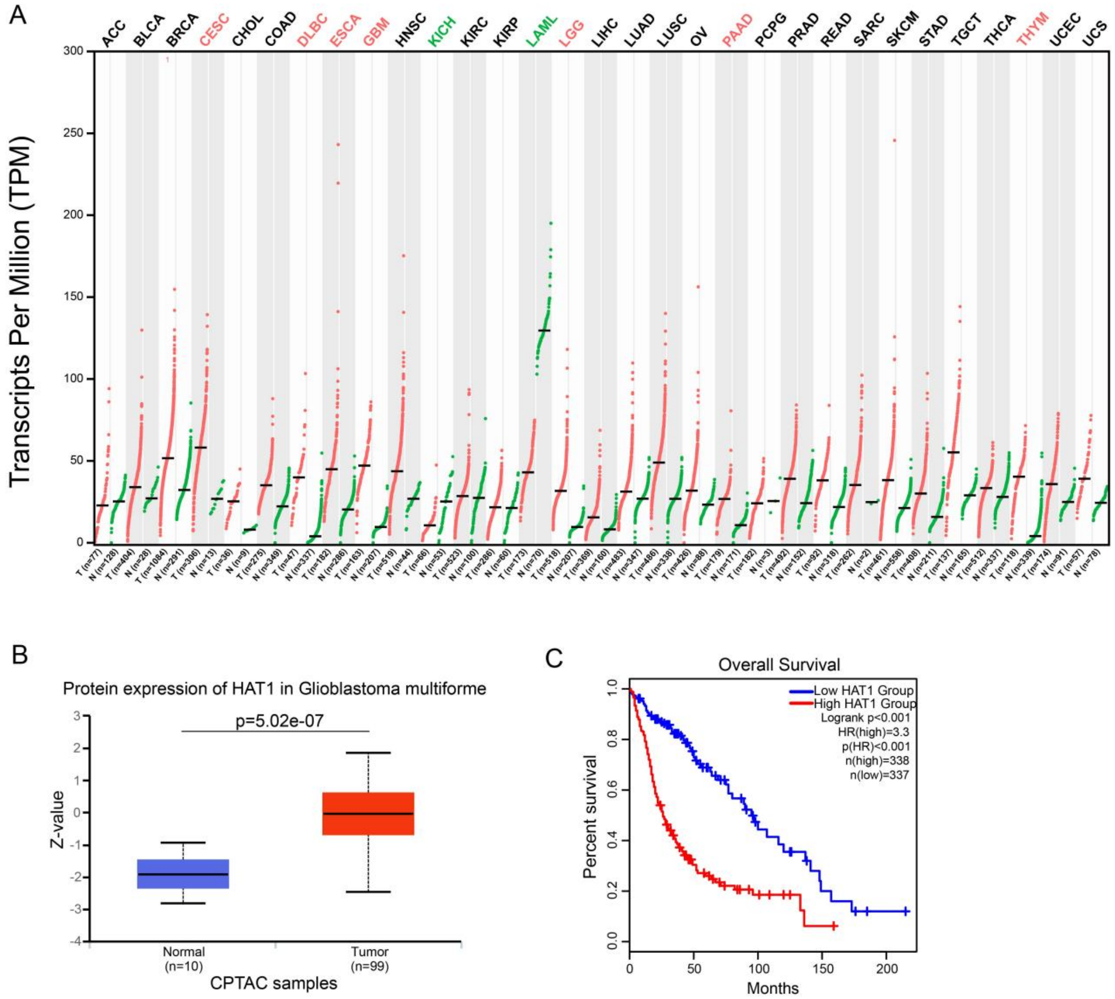

Fig. S1 HAT1 is a promising biomarker for glioma. (A). The level of HAT1 across various human tumor types and their corresponding normal samples. **(B).** Comparison of HAT1 expression between normal brain tissues and GBM samples. **(C).** Analysis of the correlation between HAT1 expression and prognosis of human glioma patients. All data were analyzed using two-way analysis of variance (ANOVA).

Fig.S2

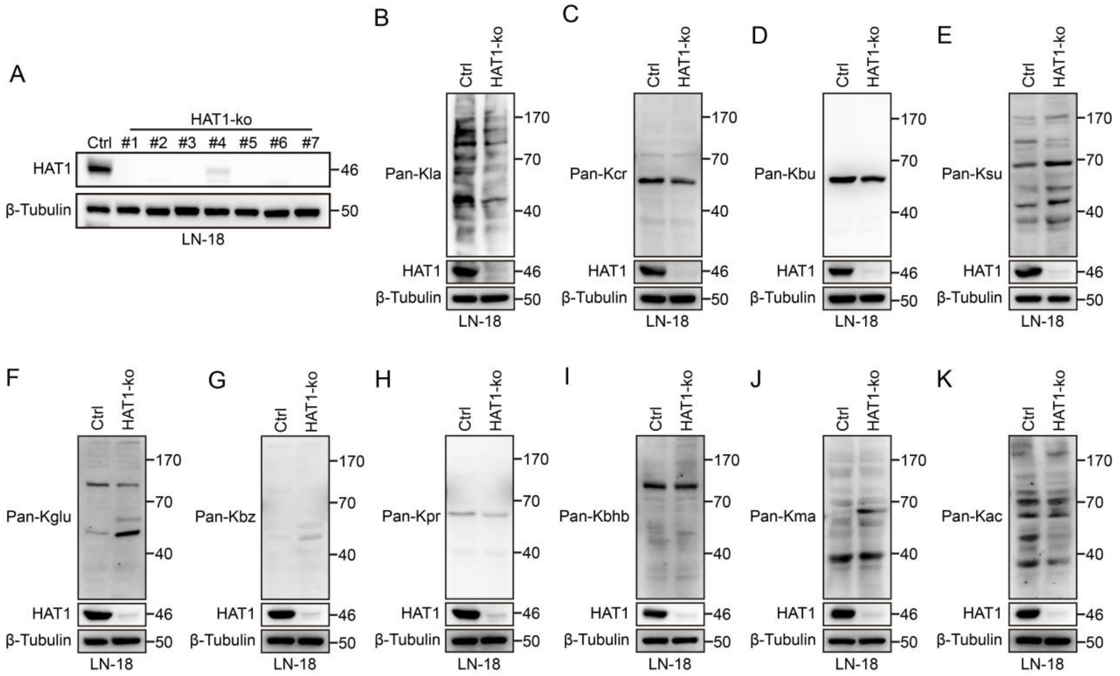

Fig. S2 HAT1 is required for global protein lactylation in LN-18 cells. (A). HAT1 expression in HAT1-ko LN-18 monoclonal cells. **(B).** The level of pan L-Lactyl Lysine (Pan-Kla) in HAT1-ko cells. **(C).** The expression of pan Crotonyllysine (Pan-Kcr) in HAT1-ko cells. **(D).** The level of pan Butyryllysine (Pan-Kbu) in HAT1-ko cells. **(E).** The expression of global Succinyllysine (Pan-Ksu) in HAT1-ko cells. **(F).** The level of global Glutaryllysine (Pan-Kglu) in HAT1-ko cells. **(G).** The expression of global Benzoyllysine (Pan-Kbz) in HAT1-ko cells. **(H).** The level of pan Propionyllysine (Pan-Kpr) in HAT1-ko cells. **(I).** The expression of global β-Hydroxybutyryllysine (Pan-Kbhb) in HAT1-ko cells. **(J).** The level of global Malonyllysine (Pan-Kma) in HAT1-ko cells. **(K).** The expression of global Acetyllysine (Pan-Kac) in HAT1-ko cells.

Fig.S3

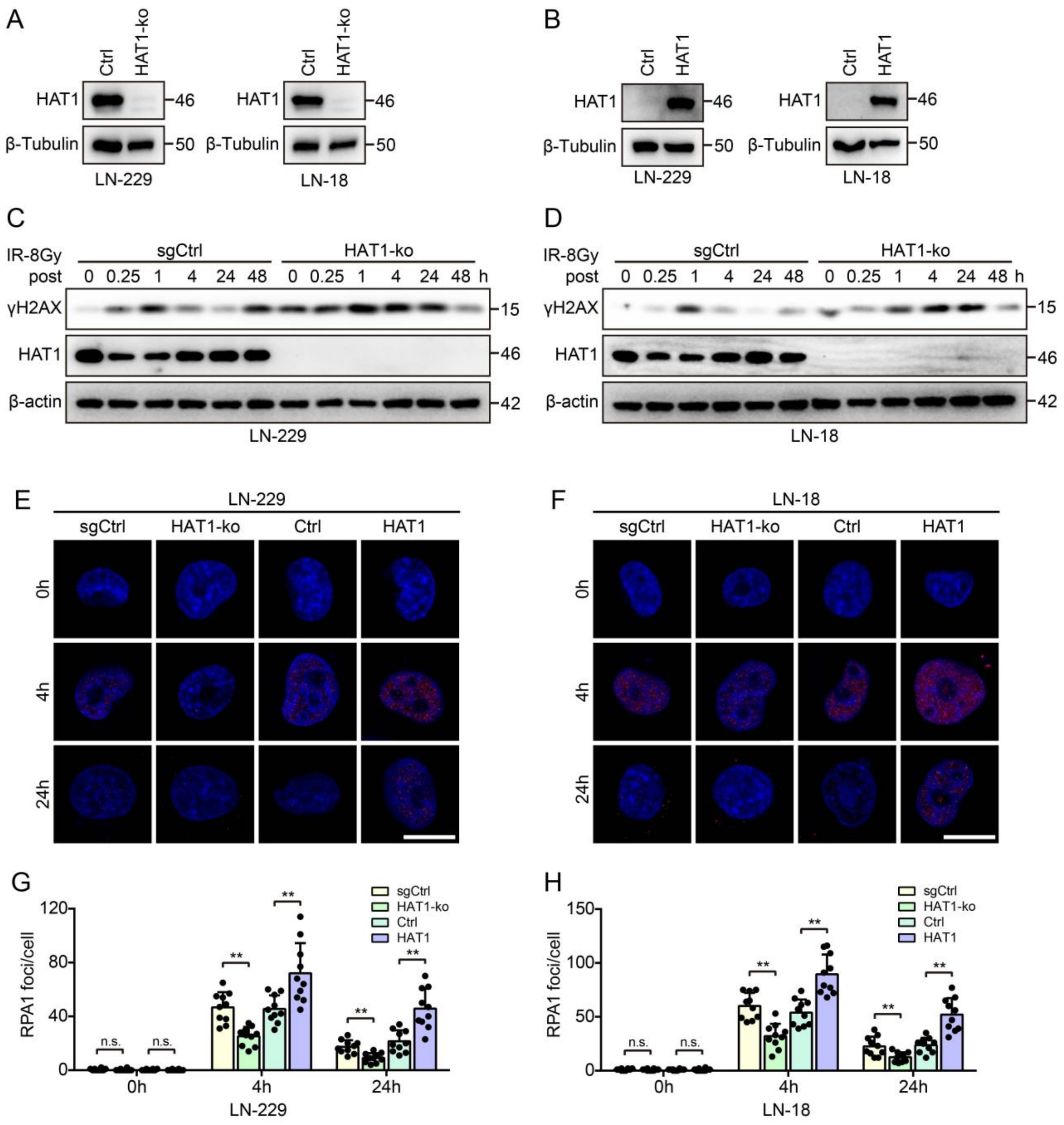

Fig. S3 HAT1 promotes DDR and radioresistance by HR in GBM cells. (A). HAT1 expression in HAT1-ko GBM cells. **(B).** HAT1 expression in GBM cells overexpressing HAT1. **(C).** Immunoblot analysis of γ-H2AX expression in HAT1-ko LN-229 cells. **(D).** Immunoblot analysis of γ-H2AX in HAT1-ko LN-18 cells. **(E).** Representative immunofluorescence images of RPA1 in HAT1-ko and HAT1-overexpressing LN-229 cells, scale bar, 10 μm. **(F).** Representative immunofluorescence images of RPA1 in HAT1-ko and HAT1-overexpressing

LN-18 cells, scale bar, 10 μm. **(G).** Quantification of RPA1 foci in panel E. **(H).** Quantification of RPA1 foci in panel F. All data were analyzed using two-way analysis of variance (ANOVA), **P < 0.01.

Fig.S4

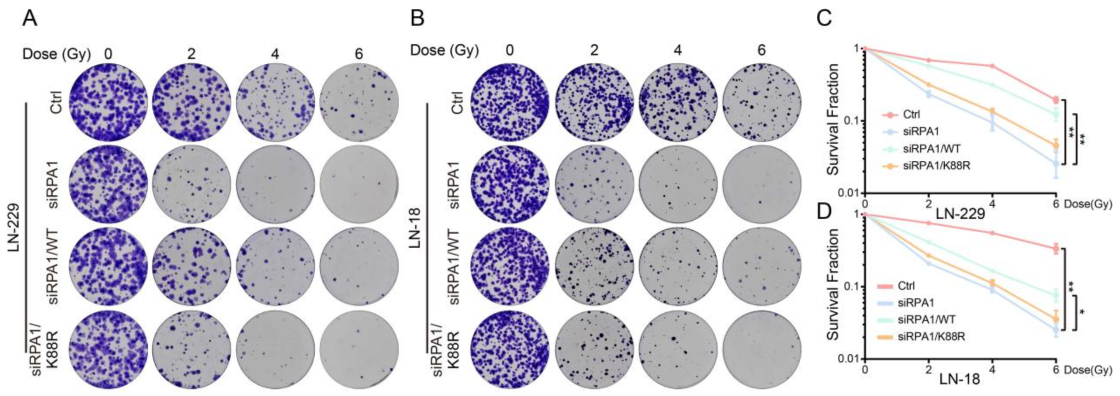

Fig. S4 RPA1 K88 lactylation is required for radioresistance in GBM cells. (A). The clonogenic ability of RPA1 K88R mutant LN-229 cells exposed to the indicated doses of IR. **(B).** The clonogenic ability of RPA1 K88R mutant LN-18 cells exposed to the indicated doses of IR. **(C).** Quantification of colony formation in panel A. **(D).** Quantification of colony formation in panel B. All data were analyzed using two-way analysis of variance (ANOVA), **P < 0.01.
